# Vegetable Salad Improves Lipid and Glucose Metabolism and Enhances Absorption of Specific Nutrients in Vegetables

**DOI:** 10.3390/foods13223591

**Published:** 2024-11-10

**Authors:** Diah Mulyawati Utari, Indri Kartiko-Sari, Toshitaka Miyazaki, Hiroko Umezawa, Yumi Takeda, Mariko Oe, Wei Wang, Sumiko Kamoshita, Motomi Shibasaki, Ryosuke Matsuoka, Shigeru Yamamoto

**Affiliations:** 1 Department of Public Health Nutrition, Faculty of Public Health, Universitas Indonesia, Depok 16424, Indonesia; utari.diahmulya@gmail.com; 2 Asian Nutrition and Food Culture Research Center, Jumonji University, Niiza 352-8510, Japanyamamotoshigeru426@gmail.com (S.Y.); 3 Jakarta Sales Office, PT Kewpie Indonesia, Jakarta Selatan 12520, Indonesia; toshitaka_miyazaki@kewpie.co.jp; 4 R&D Division, Kewpie Corporation, Tokyo 182-0002, Japan; hiroko_umezawa@kewpie.co.jp (H.U.); yumi_takeda@kewpie.co.jp (Y.T.); mariko_oe@kewpie.co.jp (M.O.); yui_ou@kewpie.co.jp (W.W.); 5 Department of Food and Nutrition, Jumonji University, Niiza 352-8510, Japan; sumiko-k@jumonji-u.ac.jp (S.K.); motomi48@jumonji-u.ac.jp (M.S.)

**Keywords:** vegetable, mayonnaise, dressing, lifestyle-related disease, postprandial blood sugar, cholesterol, carotenoid, calcium, dietary fiber

## Abstract

Vegetables are low in energy and rich in vitamins, minerals, and dietary fiber; various health benefits associated with their intake have been reported. Salads are one of the most convenient ways to consume vegetables and can be made simply by pouring mayonnaise, dressing, olive oil, or other condiments over a selection of vegetables. There are also many reports on the ways in which vegetable intake can improve health. However, there is no comprehensive review summarizing the health functions of vegetables when consumed as a salad. The effects of vegetable salads on amounts of vegetable intake, lifestyle-related diseases, and the absorption of specific nutrients through mayonnaise, as well as the effects of the order in which vegetable salad and carbohydrates are consumed, have been reported. In this review, the health functions of vegetable salad consumption are reported.

## 1. Introduction

Vegetables are herbaceous plants used in various culinary preparations. They are indispensable foods because they are rich in vitamins, minerals, dietary fiber, and phytochemicals necessary for human survival [1].

As the dietary habits of humans have diversified, the prevalence of lifestyle-related diseases and other diseases has increased; therefore, improving dietary habits is important for maintaining and promoting health [2]. One of the possible causes of poor dietary habits is low vegetable intake. It is recommended that Japanese people consume 350 g of vegetables daily, but the vegetable intake of Japanese adults is only ~280 g [3,4]. Although a vegetable intake of 250 g is recommended for Indonesian people, the average daily intake in 2020 was 143 g, falling short of the recommended vegetable intake in both countries [5]. The Indonesian Health Survey in 2023 showed that 96.7% of people consume less fruit and vegetables than recommended [6].

**Figure 1:**
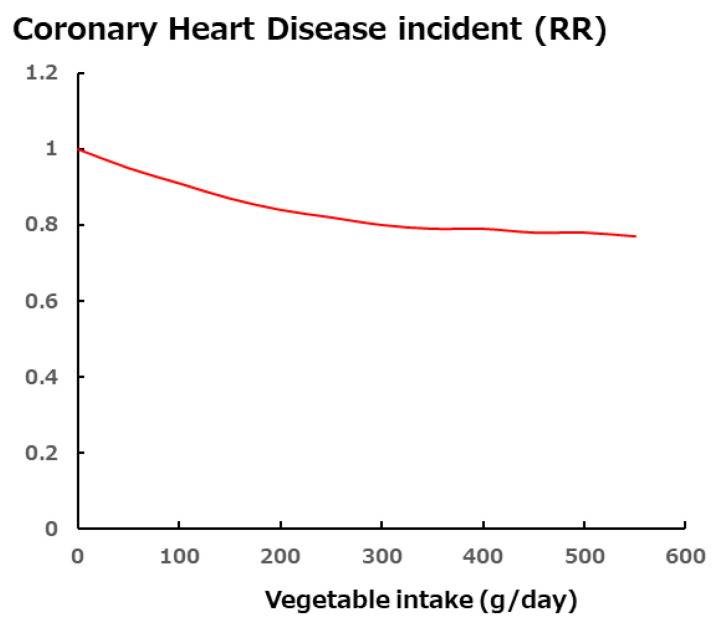
Correlation between vegetable intake and coronary heart disease incidence [7].

Increased vegetable intake reduces body weight, metabolic syndrome, and the risk of coronary artery disease (Figure 1) [7]. Metabolic syndrome is a complication caused by increased visceral fat, irregular diet, excessive food intake, and a lack of exercise and causes glucose intolerance, hypertension, and dyslipidemia, which are risk factors for arteriosclerotic illness [8]. The consumption of vegetable fiber reduces the risk of these diseases by reducing carbohydrate and lipid absorption [9,10,11]. Because vegetables also contain potassium, which improves hypertension [12,13], vegetable intake may help prevent metabolic syndrome-related diseases. Low-density lipoprotein (LDL) oxidation via reactive oxygen species is one of the causes of atherosclerotic illnesses [14], and vegetables are rich in antioxidant components such as polyphenols and vitamin C [15]. A reduced risk of bone fractures [16,17] and improved cognitive function [18,19,20] have been reported to be associated with vegetable and fruit consumption. In Japan, factors contributing to the need for nursing care include metabolic syndrome, frailty, and dementia [21]. The difference between healthy and average life expectancies is ~10 years in many Asian countries, meaning that long-term care is necessary for that duration [22]. One of the reasons for this difference is low vegetable intake. Such intake is expected to contribute to an increase in healthy life expectancy.

**Figure 2:**
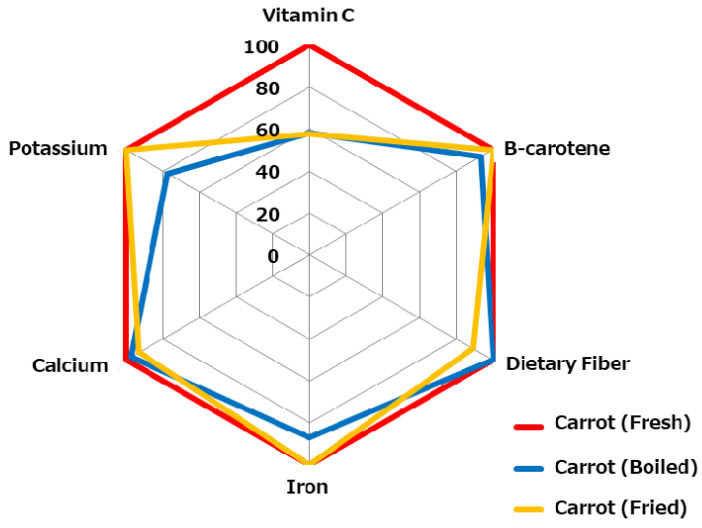
Changes in the nutrients in carrots (fresh) during cooking, set as 100% [24].

Although vegetables promote health, they are difficult to eat due to their flavor and physical properties and often require preparation to be palatable [23]. However, depending on the cooking method, the nutrients they contain may be reduced. For example, vitamin C in carrots decreases during heating (Figure 2) [24].

Salads are a simple and delicious way to consume vegetable nutrients by pouring mayonnaise, dressing, olive oil, or other seasonings over vegetables [25]. In this review article, we summarized the health benefits of consuming vegetable salads, especially those that can contribute to improving outcomes regarding lifestyle-related disease and enhance the absorption of the nutrients in vegetables.

## 2. Improvement in Body Weight and Serum Cholesterol Concentration Through Vegetable Salad Consumption

**Figure 3:**
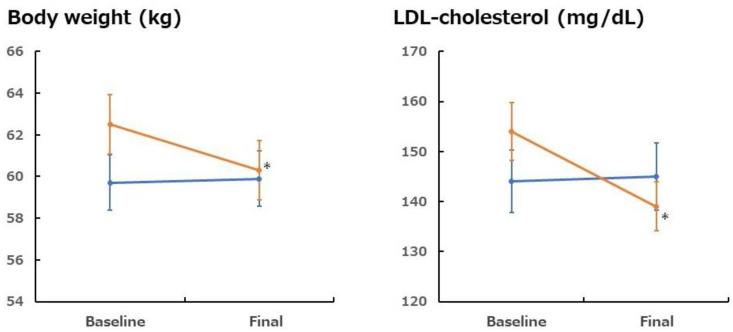
Effects of 400 g/day vegetable intake on body weight and LDL cholesterol level in Indonesian women [26]. Orange: control group (normal diet); blue: vegetable diet. Mean ± SEM (standard error of the mean) of 30 subjects. *: *p* < 0.05 vs. before intake.

Health problems associated with poor dietary habits, such as obesity, metabolic syndrome, diabetes, hypertension, stroke, and coronary heart disease, are becoming more serious. The dietary fiber intake among Indonesian people is 10 g, which is quite low [26]. A lack of fiber is a risk factor for metabolic syndrome [27]. This study considered the possibility that this is due to inadequate fiber intake (i.e., insufficient vegetable intake). A previous study examined the effects of consuming 400 g of vegetables daily [26]. The study was a parallel group comparison trial in which 60 postmenopausal Indonesian women were randomly paired into two groups (vegetable and control groups). Subjects consumed 400 g of vegetables and 20 g/200 g vegetables with mayonnaise, roasted sesame dressing, traditional peanut sauce, or chili sauce daily for 21 days. We served 400 g of vegetables daily in a salad that varied from day to day. Daily variations included three of the following vegetables: carrot, green beans, cucumber, corn, cauliflower, tomato, lettuce, cabbage, pumpkin, beansprouts, mustard greens, Chinese cabbage, broccoli, squash, and chayote. The results showed significant reductions in the total cholesterol (TC), LDL cholesterol, high-density lipoprotein (HDL) cholesterol, body weight, and body mass index (BMI) in the vegetable group compared to the baseline (*p* < 0.05). After 21 days, there was a significant decrease in energy, lipid, and carbohydrate intake (*p* < 0.05) and a significant increase in vegetable and fiber intake (*p* < 0.05). The changes in the LDL cholesterol and body weight are shown in Figure 3 [26]. These results indicated that consuming a healthy diet that includes 400 g of vegetables daily may improve diet, weight management, and lipid profiles

The above study reported a decrease in the incidence of coronary artery disease as vegetable intake increased, as shown in Figure 1 [7], and the consumption of 300 g of vegetables per day was found to reduce the risk of coronary artery disease by 20% compared to no consumption, but the risk remained, even with a higher intake [7]. Increasing vegetable intake has been reported to improve metabolic syndrome in children, adults, and elderly persons [28,29,30,31].

A study on the relationship between vegetable intake and body weight reported an inverse correlation between vegetable intake and body weight [32].

Although the above study examined a salad intake of approximately 400 g/day, it suggests that using mayonnaise or dressing to increase vegetable intake may be effective in preventing metabolic syndrome.

## 3. Inhibition of Postprandial Blood Sugar (PPBS) Levels Increases by Consuming Vegetable Salad Before Rice

Consuming vegetable salad before carbohydrates moderates the fluctuation of PPBS concentrations and is the so-called “vegetable-first” effect [33,34,35]. Considering that the effect of eating vegetables first is the effect of dietary fiber, this study also evaluated the effect of vegetable salad extract (i.e., with solids removed) consumed before carbohydrates on PPBS concentrations [36].

**Figure 4:**
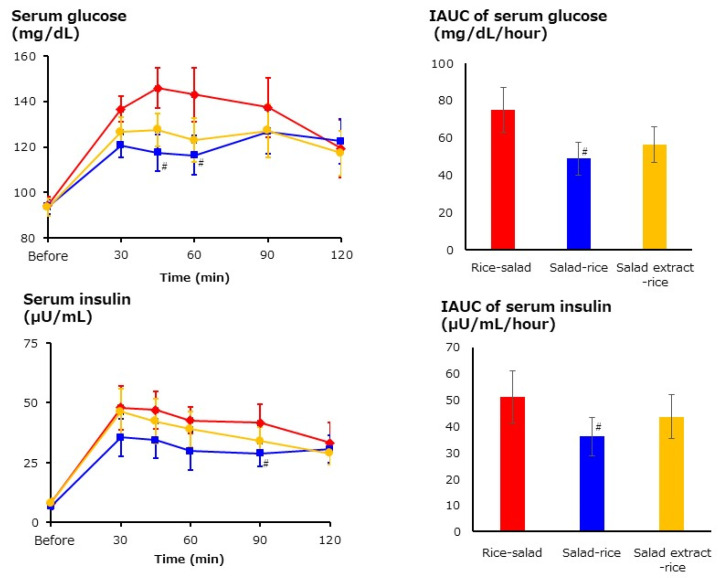
Postprandial serum glucose and insulin levels and their IAUCs (incremental areas under the curve) in subjects fed 3 types of diets [36]. ■: vegetable salad–rice (blue), ●: vegetable salad extract–rice (yellow), ◆: rice–vegetable salad (red). Mean ± SEM of 13 subjects. ^#^ Significant difference vs. rice–vegetable salad group based on Wilcoxon signed-rank test and Bonferroni correction.

A single intake crossover method was used for 13 healthy males aged >20 (washout of 1 week). The subjects had their blood sampled while hungry after an overnight fast and were asked to consume meals in three different orders: rice–vegetable salad, vegetable salad–rice, and vegetable salad extract–rice. The subjects were fed 150 g of rice, 175 g of cabbage-based vegetable salad, and 45 g of roasted sesame dressing. Blood samples were taken 30, 45, 60, 90, and 120 min after the consumption of the test diet to measure serum glucose and insulin concentrations (Figure 4). The serum glucose concentrations were significantly lower in vegetable salad–rice than rice–vegetable salad at 45 and 60 min. No significant differences were found between vegetable salad extract–rice and vegetable salad–rice or rice–vegetable salad. The serum insulin levels were significantly lower in vegetable salad–rice than rice–vegetable salad at 90 min. No significant differences were found between vegetable salad extract–rice and vegetable salad–rice or rice–vegetable salad. The results indicated that the consumption of vegetable salad before rice suppresses the increase in serum glucose concentration. The vegetable-first effect could not be explained solely by the effect of dietary fiber, as the vegetable salad extract reduced increases in blood glucose and insulin levels, although the effect was weak. As a consideration, in addition to the effect of dietary fiber, the effects of polyphenols, dressing (oil and vinegar), and chewing may have combined to suppress the increase in blood glucose levels [37,38,39].

In a recent report, it was shown that consuming potato salad before a rice meal suppresses the increase in PPBS levels [40]. The effect of cooking and cooling on the potato salad increased the amount of resistant starch, confirming that potato salad suppresses the increase in PPBS levels compared to steamed potatoes [41].

## 4. Increased Vegetable Intake and Improved Diabetes Indicators with Mayonnaise and Dressing Use

In Vietnam, an important factor in type 2 diabetes is not obesity but rather low vegetable and fiber intake [42,43,44,45]. Dietary fiber helps control blood glucose levels and can be used in the diets of people with diabetes. Vegetables are the main source of fiber in Vietnamese cuisine, but their intake is very low. This study attempted to increase vegetable and fiber intake with dressings and mayonnaise and evaluated their effects on glucose metabolism [46]. Sixty people with type 2 diabetes were randomly divided into intervention and control groups by creating 30 pairs according to their gender, age, BMI, and years of type 2 diabetes. Both groups received basic nutrition education on vegetables. The intervention group was further instructed to use roasted sesame dressing and egg yolk-type mayonnaise (the usage of the mayonnaise and dressing was not known, as the subjects were allowed to consume freely).

The study period was 2 weeks. The control and intervention groups had 300 and 450 g vegetable intake, respectively. At the end of the study, blood fructosamine levels in the intervention group were significantly lower than before the intake (*p* < 0.05) but not in the control group (*p* > 0.05).

Although this study did not specify the type of vegetables consumed, the fiber intake was about 8 g in the control group and 12 g in the intervention group [46]. Some agents found in GLV may cause a decrease in the incidence of cardiovascular diseases (CVDs). The first meta-analysis study focusing solely on GLVs (cruciferous vegetables such as cauliflower, cabbage, cress, bok choy, broccoli, kale, collard greens, and similar GLVs and their roots) found that high daily GLV intake significantly reduces the incidence of several CVDs [47]. GLV consumption decreases bile acid recirculation, utilizing cholesterol to synthesize bile acids, reducing fat absorption, and ultimately decreasing the risk of heart diseases [48]. GLVs also contain nitrates that, when consumed regularly, produce sufficient blood and tissue levels of nitrite and nitric oxide groups to compensate for disturbances in endogenous nitric oxide synthesis [49]. GLVs are also high in magnesium, which can lower the risk of CVD [50]. Molecular studies have shown that trace elements and some antioxidants in GLVs lower the risk of cancer through mechanisms that modulate free radical attack on nucleic acids, proteins, and polyunsaturated fatty acids [51].

## 5. Mayonnaise Promotes Carotenoid Absorption 

**Figure 5:**
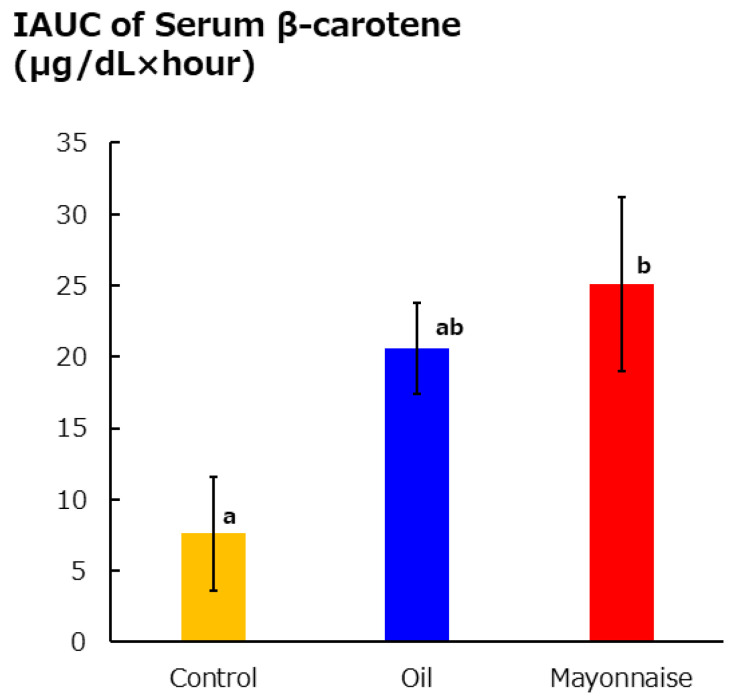
IAUC of serum β-carotene level in subjects fed 3 types of diet [53]. Yellow: carrot; blue: carrot + vegetable oil; red: carrot + mayonnaise. Mean ± SEM of 19 subjects. Different letters show a significant difference with Tukey’s test.

Carotenoids in carrot and broccoli are difficult to absorb without modification [52,53]. Because mayonnaise is often consumed with vegetables, studies have been conducted on mayonnaise consumption and vegetable nutrient absorption. Egg yolk-type mayonnaise and broccoli (boiled) together enhance lutein and zeaxanthin absorption compared to broccoli consumed on its own [52]. Egg yolk-type mayonnaise and edible vegetable oils and lipids used in mayonnaise increase postprandial serum β-carotene concentrations compared to carrots consumed alone (Figure 5) [53]. It is thought that β-carotene cannot be synthesized in the human body [54], so β-carotene levels in the blood are derived from dietary sources, indicating that mayonnaise enhances carotenoid absorption.

Regarding the absorption-enhancing effect of mayonnaise consumption on carotenoids, in vitro studies have shown that mayonnaise enhances β-carotene dissolution into the oil layer compared to oil alone in the gastric model. This is due to the emulsifying effect of egg yolk (especially egg yolk lecithin) in mayonnaise [55], and sweet mayonnaise with added sugar enhances β-carotene absorption from fruits [56]. When moderately hypercholesterolemic Japanese men ate one chicken egg daily, their serum lutein and zeaxanthin concentrations increased and LDL oxidation was inhibited [57].

Considering the above, as the carotenoid absorption enhancement effect of egg yolk-type mayonnaise is due to the effect of egg yolk and vegetable oil, carotenoid absorption is greater with mayonnaise than with vegetable oil [55]. It has also been surmised that this effect could be obtained with other types of mayonnaise, such as the whole-egg type.

## 6. Calcium Absorption Enhancement Using Mayonnaise

Although vegetables contain a large amount of calcium, it is not well absorbed due to oxalic acid, which inhibits calcium absorption and other factors present in vegetables [58,59,60,61]. It has been reported that, after dairy products, the second largest source of calcium intake among Japanese people is vegetables [4]. Spinach, in particular, is known to be high in oxalic acid [62].

Because mayonnaise is an acidic food that contains vinegar, the calcium in the vegetables dissolves into acetic acid and increases absorption [62]. Therefore, this study evaluated the effect of mayonnaise on calcium absorption in rats. This calcium absorption-enhancing effect was higher than that of acetic acid [63].

The vinegar contained in mayonnaise influences the calcium absorption-promoting effect of mayonnaise. Acetic acid, the main ingredient in vinegar, can dissolve calcium. However, because mayonnaise enhances calcium absorption more than vinegar, it is assumed that it contains ingredients other than vinegar that enhance calcium absorption. The active ingredients in mayonnaise are not clear and will be the subject of future studies.

## 7. Effect of Serum Cholesterol Concentrations of Mayonnaise Consumption

Mayonnaise is made from vegetable oils, egg yolk, and acetic acid. In Japan, its ingredients are strictly regulated by the Japanese Agricultural Standards [64]. Dressings and creamy salad dressings are generally referred to as “mayonnaise-type” products [64]. Mayonnaise is mainly affected by edible vegetable oil because of its high vegetable oil content. Edible vegetable oils commonly used in mayonnaise are soybean or rapeseed oil, which contain high amounts of oleic and linoleic acids [24]. Because these oleic and linoleic acids lower serum cholesterol levels [65], it is possible that mayonnaise could also lower serum cholesterol. In contrast, the raw material contains egg yolks. The recommended daily intake of 15 to 20 g of egg yolk-type mayonnaise contains 22.5 to 30 mg of cholesterol [24,52]. The effects of mayonnaise consumption on serum cholesterol in healthy subjects or dyslipidemic patients are summarized in Table 1. This shows that the intervention with mayonnaise that contains egg yolk and soybean oil could decrease TC and LDL cholesterol levels. Soybean oil is an emulsifier used in mayonnaise to increase its viscosity and stability [66].

In healthy subjects, consuming 15 g of egg yolk-type mayonnaise (rapeseed oil-based) daily for 12 weeks did not affect serum TC or LDL cholesterol concentrations [67]. A 70-day study of healthy Chinese youths who ate 15 g of egg yolk-type mayonnaise (soybean oil) daily resulted in a significant increase in serum TC concentrations compared to the baseline, but only by 2.9%. It did not affect LDL cholesterol concentrations [68]. Since seasonal variations in serum total cholesterol concentrations have been reported to be 3.9 mg/dL in men [69], we consider this to be a non-problematic increase [69]. In hypercholesterolemic subjects, consuming 15 g of egg yolk-type mayonnaise (rapeseed oil-based) daily for 12 weeks significantly reduced serum TC and LDL cholesterol concentrations compared to the baseline [70]. When Malaysian hypercholesterolemia subjects were given palm or soybean oil-based egg yolk-type mayonnaise in a 4-week crossover study, palm and soybean oil-based mayonnaise lowered serum TC and LDL cholesterol concentrations. The degree of decline was greater for soybean oil-based mayonnaise [71]. In a study in which hypercholesterolemic subjects consumed whole-egg mayonnaise (as a control group for a phytosterol ester-containing mayonnaise-type study), total serum cholesterol and LDL cholesterol concentrations were not affected [72]. It is unclear why whole-egg mayonnaise did not lower serum TC and LDL cholesterol concentrations. Egg white protein lowers serum cholesterol concentrations. However, because the effect can be expected with at least ~8 g of egg white protein ingested daily [73], the effect cannot be expected by the small amount contained in mayonnaise. Cholesterol-lowering effects have also been reported for egg yolk lecithin, but the amount in mayonnaise is so small that it has been thought to have no effect [74,75]. The effect of mayonnaise on serum cholesterol concentrations is the result of edible vegetable oil, but the study did not appear to show any effect of edible vegetable oil. However, the details of the type of edible vegetable oil are unknown because it is not mentioned in the study [72]. Based on the above, 15 to 20 g of mayonnaise daily does not affect serum TC and LDL cholesterol concentrations in healthy and dyslipidemic subjects and can be safely consumed.

In recent years, there have been mayonnaise-type dressings that do not use eggs [66,76,77,78,79], mayonnaise made with egg yolks from which cholesterol has been removed with supercritical CO_2_ fluid [80], and mayonnaise-type dressings containing plant sterols that lower blood cholesterol levels [81,82,83]. For those concerned about the energy content of mayonnaise, low-energy mayonnaise-type dressings are available [84,85]; for those concerned about blood pressure, mayonnaise made with flaxseed oil, rich in α-linolenic acid, which lowers blood pressure, is also available [86,87].

**Table 1:** The effects of mayonnaise consumption on serum cholesterol in healthy subjects or dyslipidemic patients.

Study	Subjects	Test Mayonnaise	Design	Duration	Main Outcome (% vs. Before Intake)	Reference
Tohgi N et al. (1997)	Healthy (*n* = 9)	Egg yolk type Canola oil base 15 g/day	Open trial	12 weeks	TC: No change LDL-C: No change	[67]
Xu WB et al. (2012)	Healthy (*n* = 47)	Egg yolk type Soybean oil base 15 g/day	Open trial	70 days	TC: 2.9% LDL-C: No change	[68]
Matsuoka R et al. (2001)	Mildly hypercholesterolemic (*n* = 10)	Egg yolk type Canola oil base 15 g/day	Open trial	12 weeks	TC: −6.3% LDL-C: −8.4%	[70]
Karupaiah T et al. (2016)	Normal and mildly hypercholesterolemic (*n* = 34)	Egg yolk type Soybean oil or palm oil 20 g/day	Cross-over	4 weeks	Palm oil base: TC: −2.7% LDL-C: −4.5% Soybean oil base: TC: −7.7% LDL-C: −9.5%	[71]
Ishizaki T et al. (2003)	Normal and mildly hypercholesterolemic (*n* = 29)	Whole-egg type Oil unknown 15 g/day	RCT	3 months	TC: No change LDL-C: No change	[72]

TC: total cholesterol; LDL-C: LDL cholesterol; RCT: randomized controlled study.

## 8. Discussion

This review showed that vegetable salad increases vegetable intake, improves lipid and glucose metabolism, and enhances carotenoid and calcium absorption from vegetables.

Fruit and vegetable consumption is associated with decreased all-cause mortality and reduced cancer and cardiovascular mortality. Vegetables may have a stronger association with mortality than fruits [88].

In terms of preventing cardiovascular mortality, the effects of vegetable salad consumption on weight loss and metabolic syndrome prevention appear to be due to dietary fiber. As dietary fiber remains in the stomach longer [89], vegetable consumption is expected to make people feel full and reduce the intake of meals high in carbohydrates and lipids. Dietary fiber suppresses carbohydrate and lipid absorption. There are also benefits of chewing and consuming vegetables. Regarding the effect of mastication, cabbage consumption increases the number of times one has to chew compared to rice, boiled eggs, fish, and sausages [90]. The more chewing is performed, the more saliva is secreted [91]. Vegetable dietary fiber can hold water; when mixed with saliva through chewing, it becomes gelatinous and viscous [92]. The high viscosity of gastric contents slows carbohydrate absorption [93]. Epidemiological studies that examined the relationship between chewing ability and diabetes reported that groups with a strong chewing ability had a reduced risk of diabetes compared to groups with a weak chewing ability [94]. More frequent chewing increases insulin secretion and suppresses increases in PPBS concentrations [95]. The consumption of olive oil and rice together suppresses increases in PPBS concentrations in a concentration-dependent manner, although the difference is insignificant [38].

In terms of cancer, a study in Italy showed that the dietary pattern of salad vegetables had a protective effect against HER (human epidermal growth factor receptor)-2-positive cancers, much stronger than HER-2-negative cancers [96]. An important finding is that the dietary pattern of salad vegetables protects mainly against a specific breast cancer subtype [96]. A study in the United States showed that soluble CD44 (cluster of differentiation 44), a simple and inexpensive marker of cancer stem cells, increased in oral cancer patients who ate more green salad [97]. This was the first study to show that increased green salad intake is associated with improved progression-free and overall survival and lower CD44 levels in mouthwash from oral cancer cases with long-term follow-ups. Eating salad is one way for patients to increase vegetable consumption to prolong their survival [97]. Furthermore, dietary fiber reduces the risk of gastric, colorectal, and breast cancers, suggesting that vegetable consumption may help prevent cancer [98,99,100,101,102]. In addition, vegetables have been reported to contain many other bioactive substances and nutrients. Dietary fiber, abundant in vegetables, improves bowel movements, and the same effect can be seen in the consumption of vegetable salads [103,104]. Dietary fiber is known to improve the intestinal environment [105]. It has been reported that dietary fiber improves the intestinal environment, upgrades the colonic mucus barrier, and enhances immune function, thereby reducing susceptibility to pathogens [106,107]. In addition, antioxidant components such as polyphenols and carotenoids, which are included in vegetables, have also been reported to improve the intestinal environment [108,109]. Rocket salad also contains lactic acid bacteria, which have been reported to improve the intestinal environment [110,111]. However, the effect of vegetable salad consumption on improving the intestinal environment has not been reported; therefore, we will expect this to be investigated in future studies.

Vegetables contain a lot of antioxidant components such as carotenoids, vitamin C, vitamin E, and polyphenols (flavonoids, etc.) [24,112]. It has also been reported that salad seasonings such as herbs and vinegar have antioxidant capacities [112]. In this review, we have shown that mayonnaise promotes carotenoid absorption from carrots and broccoli [52,53]. For this reason, it is considered that antioxidant activity in humans is also caused by the ingestion of antioxidants as a salad. It has also been reported that a high intake of vegetables reduces oxidative stress in humans [113]. Because oxidation in vivo has become a risk factor for arteriosclerotic diseases, cancer, and Alzheimer’s disease [14,114,115], salad is considered to have the potential to prevent these diseases.

Vegetables also contain many other nutrients. In Japan, numerous foods with function claims that are ingredients contained in vegetables and used as active ingredients have been submitted and accepted [116]. Table 2 summarizes the ingredients related to vegetables extracted from the search results for “fresh foods” on the foods with function claims search site [116]. Since functional foods in Japan can be submitted with evidence of active components, it is necessary to confirm the effects of vegetables per se in the future, since RCTs are often not conducted on the vegetables themselves. Furthermore, a subject for future study would involve determining how the function of such vegetable components is altered when incorporated into a salad.

In an American study, subjects who ate salads had a better intake of dietary fiber, total fat, unsaturated fatty acids, vitamins A, B6, C, E, and K, folate, choline, magnesium, potassium, and sodium [117]. The number of subjects was significantly higher for the total food ingredients of vegetables, green vegetables, nuts, whole fruits, total protein foods, seafood, vegetable proteins, fatty acids, refined grains, and added sugars [117]. The consumption of salads, raw vegetables, and salad dressings was positively associated with increased levels of the following serum micronutrients: folic acid, vitamins C and E, lycopene, and α- and β-carotene. Each serving of salad had a vitamin C content of >100% of the dietary recommendations [118].

Overall, vegetable salad products can be an alternative healthy food [119]. Based on the above, although salad is a food that contains diverse ingredients, the dietary fiber in vegetables and the vinegar and edible vegetable oils and lipids contained in mayonnaise and dressings contribute mainly to health functions.

The results of a study conducted on Indonesian students showed that vegetable salad is well received in terms of taste, aroma, and presentation [119]. Although vegetable intake is recommended in many countries, it is likely that the intake does not meet the recommended amounts in many cases. Although vegetables contain components involved in health promotion, it is assumed that their intake may be avoided due to the complexity of their preparation and their flavor [120].

Salads are easy to prepare and a tasty way to consume vegetables. However, there may be cases where raw vegetables cannot be consumed for safety reasons. In this case, using mayonnaise or dressing on warm vegetables can also be a tasty and healthy way to consume vegetables.

Vegetable intake alone does not provide protein, but adding eggs, cheese, or chicken to a vegetable salad does. Eggs contain many nutrients but not vitamin C and fiber, and they are thought to provide a balanced diet when consumed with salad. In contrast, because mayonnaise and dressings use edible vegetable oils and lipids as ingredients, there is an apprehension that their excessive consumption may lead to a high energy intake, so they must be used in moderation.

## 9. Conclusions

This review focuses on the functions that vegetable salads serve in terms of health, particularly those consumed with mayonnaise or dressing. It has been reported that vegetable salads function in two ways: first, increased vegetable intake is effective in terms of helping to prevent lifestyle-related diseases by improving glucose metabolism, obesity, and lipid metabolism; second, salad seasonings are effective in promoting the absorption of the carotenoids and calcium contained in vegetables.

Future research into the health functions of these functional vegetable ingredients would further enhance the health value of vegetables and vegetable salads. This review will contribute to the maintenance and improvement of health through the intake of salads.

## Figures and Tables

**Table 2:** Vegetables extracted from the search results for “fresh foods” on the foods with function claims on the search site [116].

Functional Component	Vegetables Containing Functional Ingredients	Functionality	Estimated Daily Intake of Functional Ingredients
GABA	Tomato	Function of maintaining memory and spatial cognition as part of cognitive function	100 mg
	Paprika	Function of reducing temporary stress	28 mg
	Sowing	Ability to lower blood pressure	20 mg
	Cabbage	Ability to lower blood pressure in people with high blood pressure	12.3 mg
	Broccoli		
	Paprika		
	Kale		
	Soybean moyashi		
	Bean seedling		
Soy isoflavone	Soybean moyashi	Function of helping maintain the components of bones	23.3 mg
		Function of maintaining skin moisture in middle-aged and elderly women who tend to have dry skin	30 mg
Lycopene	Tomato	Function of helping protect the skin from UV irritation	16 mg
		Ability to increase HDL cholesterol	15 mg
		LDL cholesterol-lowering function	22 mg
Inulin	Garlic, chrysanthemum, chicory, burdock root	Ability to regulate physical conditions	5 g
		Ability to reduce postprandial glucose levels	0.75 g
		Function of suppressing the elevation of blood triglycerides after meals	8.1 g
Quercetin	Coconut	Function of helping maintain a positive mood	50 mg
Sulfora glucosinolate	Broccoli	Function of lowering elevated blood levels of hepatic enzymes (ALT)	24 mg
	Broccoli sprout		
	Scale plout		
	Broccoli	Ability to increase skin moisture and relieve dryness	20 mg
	Broccoli sprout		
	Scale plout		
Corin ester (acetylcholine) from eggplant	Sowing	Ability to lower blood pressure in people with high blood pressure	2.3 mg
Xanthophylls from paprika	Paprika	Function of helping protect the skin from UV irritation	9 mg
		Function of helping reduce body fatness and improve BMI	9 mg
Lutein	Spinach	Function of protecting the eye from light-induced stimulation	10 mg
	Kale	Function of protecting the eye from light-induced stimulation	10 mg
	Pumpkin	Function of protecting the eye from light-induced stimulation	10 mg
	Komatsuna	Function of improving contrast sensitivity (clear vision to reduce blurring)	6 mg
Dietary fiber derived from tomato	Tomato	Ability to reduce postprandial glucose levels	1.6 g
		Function of suppressing the elevation of blood triglycerides after meals	1.6 g
6-gingerol, 6-gingaol	Ginger	Function of maintaining body temperature at peripheral sites	2.35 mg
β-carotene	Green/yellow vegetable	Function of reducing nasal discomfort caused by house dust or dust	4.7 mg

## Data Availability

No new data were created or analyzed in this study.

## Abbreviations

BMI	body mass index
CD	cluster of differentiation
CVD	cardiovascular disease
GLV	green leaf vegetable
HDL	high-density lipoprotein
HER	human epidermal growth factor
IAUC	incremental area under the curve
LDL	low-density lipoprotein
PPBS	postprandial blood sugar
RCT	randomized controlled study
SEM	standard error of the mean
TC	total cholesterol
